# “We felt so proud by the president calling us my heroes.” An exploration of the nurse’s experiences in the management of COVID-19 patients in Uganda

**DOI:** 10.1186/s12912-023-01503-6

**Published:** 2023-10-03

**Authors:** Faith Nawagi, Martin Lubega, Aidah Ajambo, John Mukisa, Rose Nabirye

**Affiliations:** 1 College of Health Sciences, Makerere University, Kampala, Uganda; 2 College of Health Sciences, Department of Nursing, Makerere University, Kampala, Uganda; 3 Uganda Prisons Service, Kampala, Uganda; 4 School of Biomedical Sciences, Makerere University College of Health Sciences (MaKCHS), Kampala, Uganda; 5 Faculty of Health Sciences, Department of Nursing, Bustiema University, Kampala, Uganda

**Keywords:** COVID-19, Nurses, Experiences, Uganda, Africa

## Abstract

**Introduction:**

Adequate and intensive nursing care was a key characteristic of recovery of the COVID-19 patients globally and in Uganda. However, there is limited literature on the experiences of nurses who participated in the care of COVID-19 patients in Uganda, East Africa, and Africa at large, yet imperative in designing approaches to increase the efficiency of the health systems’ response to future pandemics. To address this gap, this study aimed to explore the experiences of the nurses who managed COVID-19 patients at Mulago National Referral Hospital in Uganda.

**Methods:**

This was an exploratory qualitative study that used purposive sampling to identify 21 nurses who treated COVID-19 patients at Mulago National Referral Hospital in Uganda. Focus Group Discussions were used to collect data. Thematic Analysis was used to analyze the data. Common codes were identified and grouped to create subthemes and major themes.

**Results:**

Six themes were identified: 1) Motivation to work on COVID-19 patients, 2 ) Roles performed by nurses, 3) High workload and professional role strain, 4) Challenges with maintaining personal health and relationships, 5) Institutional and government support, 6) Acquired professional knowledge and skills to manage critical patients and epidemics. Most of the nurses faced work burnout, social isolation, stress, and psychological trauma. However, interprofessional collaboration, financial incentives, government recognition, and provision of personal protective equipment, were key motivators for the nurses. The majority reported to have gained new knowledge and skills in the management of pandemics and highly infectious diseases.

**Conclusion:**

The nurses experienced negative scenarios like work burnout due to high workload, social isolation, and psychological stress. Therefore, there is a need for health systems to develop approaches and policies that support nurses’ well-being. Nevertheless, key attributes like resilience, adaptability, and diligence to serve enabled them to persevere despite the hardships faced.

**Supplementary Information:**

The online version contains supplementary material available at 10.1186/s12912-023-01503-6.

## Introduction

The COVID-19 pandemic remains one of the biggest global health emergencies that greatly exacerbated the strain on almost all health systems around the world [1]. The strain was much greater in the already overburdened and fragile health systems in resource-constrained countries, especially in Sub-Saharan Africa [2, 3]. By 8th April 2023, the World Health Organization (WHO) had reported over 762.791 million confirmed cases with over 6.89 million deaths due to COVID-19 worldwide [4]. Recently on the 10th May 2023, WHO declared the end of the COVID-19 pandemic as a global health emergency [5].

COVID-19 being a novel disease, there isn’t any approved treatment to date and all clinical management of the COVID-19 cases was symptomatic and sorely relied on supportive treatment [6–8]. Faced with the infection’s high attack rate, and no approved treatment, many countries including Uganda adopted a range of treatment approaches and clinical guidelines to manage patients [9, 10]. Nurses were at the forefront of the pandemic response with great involvement in the extensive coordination of services, screening, vaccination, and front-line work in respiratory, emergency, and intensive care environments [11]. This nature of work was often intense and stress-provoking with an inevitable psychological impact on nurses and all healthcare workers [12]. Studies have shown that various healthcare professionals particularly nurses in many countries worked in high-stress environments with shortages of staff, experienced burnout, depression, anxiety, family and social avoidance due to stigma or fear of the disease [3, 13–17]. Lack of psychosocial support for nurses reported in some settings affected the quality of care and health of the caregivers [17].

Uganda is one of the countries that made significant progress in handling the COVID-19 pandemic. Of the 170,515 confirmed cases by 12th April 2023, only 3,632 deaths were registered with a recovery rate of over 98% [4]. Mulago Specialized Hospital which is also the national referral of the country has handled the largest number of patients in the country [18]. COVID-19 was characterized by long hospitalization with an average stay of 20.6 days by the time of discharge [19]. Adequate and intensive nursing care was a key characteristic of the recovery of COVID-19 patients [19]. However, there is limited literature on the experiences of nurses who participated in the care of COVID-19 patients in Uganda, East Africa, and Africa at large, yet imperative in designing approaches to increase the efficiency of the health systems’ response to future pandemics. A study done by Kabunga et al. in Uganda found a high prevalence of burnout caused by workload among nurses who treated COVID-19 patients [20]. However, it did not provide an in-depth all-round description of the nurses’ experiences. To address this gap, this study aimed to use a qualitative approach to explore the experiences of the nurses who managed COVID-19 patients at Mulago National Referral Hospital in Uganda. This in the long run would enable the policy makers to design approaches in response to epidemics and pandemics that enable adequate support for the frontline health workforce which mainly includes nurses.

## Methodology

### Study design

This was an exploratory qualitative study that was conducted among nurses who provided care to COVID-19 patients at Mulago National Referral Hospital in Uganda. This design was chosen because it allows a wider description of the topic allowing an in-depth understanding of the objective at hand [21]. Furthermore, the design allows flexibility in implementation and adaptation to change [21]. Uganda’s COVID-19 health emergency happened from 2020 to 2022. This study was conducted from January to May 2023.

### Study context and setting

This study was conducted at Mulago National Referral Hospital. This is Uganda’s super-specialized hospital handling complicated and advanced cases from the regional referral hospitals in the country. Given its ability to handle complex cases, the facility was selected as one of the COVID-19 treatment centers and it handled the biggest number of patients [22]. It had over 900 beds for COVID-19 management and an intensive care unit to handle any emerging COVID-19-related complications [18]. The hospital is a public, (government not-for-profit hospital) with free access to care and is located close to the country’s institutional quarantine centers thus enabling timely referral [18].

### Study population

We included nurses who had participated in providing nursing care for COVID-19 patients from 2020 to 2022. These later years were chosen because that was the time when Uganda was facing its COVID-19 health emergency. The nurses were of varying gender, age group, seniority of practice, and education levels. This allowed a diversity of opinions and sharing experiences. More than 70 nurses participated in the treatment of COVID-19 patients at Mulago National Referral Hospital. Given that this was a qualitative study of which point of saturation [23] would determine the number to be recruited, we recruited 21 participants in this study.

### Sampling method and recruitment of participants

Purposive sampling was used to identify study participants. This was used because of the nature of the study being qualitative and requiring us to get the best-fit participants to gain deeper insights into the study objectives. The criteria used for the selection of participants was that one had to have participated in providing COVID-19 nursing care to hospitalized patients at Mulago National Referral Hospital. Nurses who were not willing to provide informed consent and those who were sick were excluded from the study. More than 70 nurses participated in the treatment of COVID-19 patients at Mulago National Referral Hospital. Phone contacts of the nurses were obtained from the nurse in charge of the COVID-19 unit. Each of the eligible participants was contacted via phone call for their interest in participating in the study. Upon acceptance, an online consent form and online Zoom link were sent to them. As a result, we conducted five Focus Group Discussions (FGDs) and recruited 21 participants in total. Saturation was reached at 12 participants in the third FGD. However, two more FGDs were conducted to confirm saturation leading to 21 participants in total that were recruited. Each FGD had four participants except the fifth FGD which had five participants.

### Study tools

For establishing the experiences of Nurses, an FGD guide that was previously used to establish the experiences of nurses on Ebola management [21] was adopted since COVID-19 is also an epidemic and requires the same approaches of strict protection and constant nursing care as for Ebola. The tool was modified to suit this study as shown in Appendix 1. This tool was used simply because there were hardly any standardized tools that had been developed for this study purpose (COVID-19) in Uganda and Africa at large at the time of implementing this study. Furthermore, the tool was piloted and verified with the study team before use. The pilot was done among two nurses who had participated in the treatment of COVID-19 patients in another hospital. The suggestions for refinement were made and reviewed by the study team. The results of the pilot were not included in the main study.

### Data collection

Data were collected from January to May 2023. Participants were approached via phone call, thereafter, the consent forms and FGD guide were emailed to the participants at least 2 days before the FGD to enable substantive preparation. The FGDs were conducted via Zoom. Each FGD lasted 1.5 h and had four participants except one that had five participants. Participant responses from the FGDs were audio-recorded via Zoom and later transcribed verbatim. We conducted five FGDs; however, the point of saturation was reached at three FGDs. Saturation meant the point where no more new information was emerging during the FGD. To confirm saturation, two more FGDs were conducted. While the point of saturation determined the number of participants and FGDs in this study, efforts to ensure the nurses were of varying gender, age group, seniority of practice, and Education levels were made. This allowed a diversity of opinions and sharing experiences. The FGDs in this study were conducted virtually online using Zoom.

### Data analysis

Audio recordings were transcribed verbatim by a social anthropologist competent in English and transcription. The recordings and transcripts were examined by the research team and the principal investigator for consistency against audio files. The transcripts were repeatedly read through by the research team to ensure they were correct, and the responses were complete. Inductive Thematic analysis [24] was employed using open code software 4.03 [25]. This was done by reading the transcripts several times (3–5 times) by the qualitative data analysis expert to identify meaningful units and texts to develop codes. The codes generated from the transcripts were categorized and emerging subthemes and themes were generated. Codes that failed to fit in one subtheme but made much more meaning in another subtheme and theme were redistributed to where they made much more meaning. The themes and subthemes were read and reviewed by the research team for alignment with the study objectives. The social demographic characteristics of the participants were summarized and presented as frequencies and proportions.

### Quality control

The trustworthiness and rigor of this study given its qualitative nature were observed. For credibility, prolonged engagement of the participants during data collection, having the research team review the findings, and review by a qualitative analysis expert were done. The research team and the qualitative data expert had experience in handling qualitative data collection and analysis for more than five years and more than three qualitative studies handled by each. We also identified our own biases to avoid their interference with the research findings by applying reflexivity during data collection and analysis. This was done by recording all the FGDs, ensuring immediate transcription, taking notes during the FGDs, and continually editing our subjective statements. Furthermore, the collection of data from nurses of various social demographic characteristics was done to ensure triangulation as shown in Table 1. A detailed description of the qualitative data collection and analysis process was done to ensure transferability in similar contexts elsewhere. To observe the dependability of the findings Open Code software 4.03 [25] was used to derive findings. To observe confirmability the study findings were reviewed by the study team for accuracy and alignment with the study objectives.

## Results

### Socio-Demographic characteristics of the Nurses that participated in the study

As shown in Table 1, more than three-quarters of the nurses (18, 85.7%) had no prior experience managing an epidemic. Of the 21 participants who participated in the interviews for this study, (11,52.8%) and (10,47.6%)) were males and females respectively. Most of the participants (14, 66.7%) had attained up to a bachelor’s level of education and the same proportion was married. All the participants reported to have had access to personal protective equipment.

**Table 1:** Study participants socio-demographic characteristics N = 21

Characteristic	Frequency (n)	Percentage (%)
**Gender**		
Male	11	52.8
Female	10	47.6
**Highest level of nursing training**		
Bachelors	14	66.7
Diploma	5	23.8
Masters	2	9.5
**Marital status**		
Divorced	1	4.8
Married	14	66.7
Single	6	28.6
**Religious affiliation**		
Christianity	19	90.5
Islam	2	9.5
**Prior experience managing epidemic outbreaks in Uganda**		
No	18	85.7
Yes	3	14.3
**Access to PPEs**	21	100.0
**Number of children /dependents**		
Median, Interquartile range	2,1–4	
Minimum, maximum	1, 15	
**Years of experience**		
Median, interquartile range	9, 5–16	
Minimum, maximum	3,29	

### Experiences of the nurses managing COVID-19 patients in Uganda

Thematic analysis revealed six major themes: motivation to work on COVID-19 patients, roles performed by nurses, high workload and professional role strain, challenges with maintaining personal health and relationships, institutional and government support, and acquired professional knowledge and skills to manage critical patients and epidemics. The experiences of nurses with prior experience in managing epidemics and those without did not vary. Furthermore, there was not much difference in the nurse’s experiences despite the varying characteristics. The various themes and the corresponding quotes are displayed below.

#### Theme 1 motivation to work on COVID-19 patients

The majority of the nurses reported that the desire to save lives, passion for their jobs, financial incentives, empathy, moral duty, and high teamwork at the COVID-19 treatment units (CTUs) motivated them to join in the care of the high-risk COVID-19 patients. The nurses told us:

*“Bringing yourself to the front line to fight COVID-19 also came with a benefit. There was some allowance of ugx 80,000/= per day. That was also a motivator because some of us needed that risk allowance on top of the salary”-FGD4.*

Another participant described inner calling as a motivator:

*“I joined the COVID-19 team during the second wave after suffering from the disease in the first wave. So, I knew what it meant, the loneliness, the stigma, and the psychological aspect of it. So, according to me, attending to COVID patients was a call. Looking at how people were dying especially in the second wave, money was not a motivator for someone to work on these patients sincerely but the inner calling.”- FGD 1*.

#### Theme 2 various roles performed by the nurses

The roles performed by the nurses included drug administration, nutritional care, intense monitoring of patient vitals, positioning of patients, and psychological support. This is reflected in the quotes below.

### Drug administration

*“In regard to drugs; at the start of COVID-19 in 2020, Mulago Hospital was able to provide almost all the drugs that the patients needed, and patients were not buying any treatment. But as the numbers kept rising, we had a scarcity of some drugs like clexane which was routinely used, and the time came when patients had to buy some of the drugs. “.-FGD 1*.

#### Nutrition care

*“When it comes to nursing care, food was provided by Mulago Hospital except for the patients who had special needs. These were allowed to bring in other feeds from the outside. For patients who could not feed themselves, nurses had to sit by their bedside and feed them either through a nasal gastric tube or helping them to feed by scooping the spoon or a fork to their mouth.”-FGD 1*.

#### Intense vital signs monitoring

*“We had to ensure that we strictly monitor the patients’ vitals hourly to ensure that they are all stable. COVID-19 patients had two things that we were interested in, that is the oxygen saturation and the respiratory rate since most of them were hyperventilating and the saturations were dropping.”-FGD 1*.

### Positioning of patients

*“We had to go a step further to ensure that we position them in comfortable positions. Most of them preferred the supine position or the cardiac/ sitting position. Let us not forget what came up trending then, which was the prone position that came on board.*”-FGD 3.

### Psychological support

*“We would do counseling and give psychosocial support. Those who could pray with the patients did, gave them encouraging words.”- FGD3*.

#### Theme 3 high workload and professional role strain

Taking care of COVID-19 patients came with an increased workload for all the nurses working in the Intensive Care Unit (ICU) and wards at the hospital. This situation was exacerbated by the restrictions and lack of patient attendants, which transferred immense work to the nurses. As shown in the quotes below.

### Lack of patient attendants

*“During COVID-19 in Mulago, we didn’t have attendants to patients meaning that everything that the patient needed was to be done by the health workers. Regarding nursing care, we did everything for the patient starting from drug administration, nutrition, bed bath, oral care, and all that. So, the patient was comfortable because of the nursing care we could give.”- FGD 1*.

### Many patients to attend to

*“In ICU each nurse was caring for about five patients who all needed intensive monitoring every hour which was a bit overwhelming .”- FGD 4*.

### Long work hours

*“We were working 6 hours that is from 6 pm to 12 midnight but this changed to 4 hours after the recruitment of more nurses and teamwork. We thank the government that gave us reinforcement to enable us to work for shorter hours and effectively”- FGD 3.*

#### Theme 4 challenges maintaining personal health and relationships

The various challenges mentioned by the nurses as they cared for hospitalized COVID-19 patients included Challenges staying healthy, getting infected with COVID-19, Psychological trauma, social isolation, and psychological stress to families and friends. This is reflected in the quotes below.

### Challenges staying healthy

*“I had a problem with feeding when I was being nursed in ICU. I was always too hungry, and my metabolic rate was very fast, so I understood that when a COVID-19 patient wanted a meal or a drink, they wanted it. Remember we were giving these patients dexamethasone; the sugars were altered.”- FGD1*.

### Psychological trauma

*“For all the times I have been in ICU, I have never seen people die like the way they did so I was very traumatized that even when the Ministry of Health sent counselors, they couldn’t address my depression other than annoying me since they didn’t know what was taking place in ICU.” -FGD4.*

### Social isolation

*“When I started working in the COVID-19 unit I didn’t go back to my family until the second wave was over. They told me not to come back home until three weeks were finished.”- FGD3.*

### Psychological stress to families and friends

*“It(my working in the COVID-19 unit) put them (relatives) in tension with fear that their son, their brother was in a COVID-19 Unit where most of the patients were not going home but dying. However, they could encourage me. My grandmother was always praying for me.”- FGD 3*.

*“ My friends could also give me discouragement messages, Why did you choose to go to that unit, Why did you take yourself there when you knew it? Such comments could come in but on the other side I had some fair comments from a few colleagues, staff, and some family members though the majority were not supporting it.”- FGD 3*.

#### Theme 5 Institutional and Government Support

The nurses felt that the government offered some incentives to them for their work by classifying them as essential workers which allowed them to have access to the hospitals, financial risk allowances, and providing Personal Protective Equipment (PPE) which was key for psychological and physical safety. This is reflected in the quotes below.

### Classification as essential workers by the government

“*We must also commend the political wing for that kind of lullaby of calling us the essential workers. We felt so proud by the president calling us my heroes, my scientists made us serve the nation with more strength. Really, the political wing did a lot to push us forward and gain more strength to fight the disease.”- FGD 4*.

### Providing Personal Protective Equipment (PPE)

*“PPEs were provided. I would like to thank Mulago Hospital and the Ministry of Health. At least I can testify that in Mulago we used full PPEs from the start to the end.”-FGD1.*

#### Theme 6 acquired professional knowledge about managing critical patients and epidemics

The nurses informed us that they gained new knowledge inline to critical care, how to use Personal Protective equipment, and maintain strict adherence to the Standard operating procedures during the care of critically ill patients. This is exhibited in the quotes below.

### Learning how to use PPE

*The moment COVID = 19 came in we had to adjust to PPEs which was very challenging to the body in the beginning but later the body got used I could work with the mask without any challenge.”- FGD1*.

### Learning how to handle pandemics

“*Professionally, it has really given me an experience of how to handle a pandemic. We were taught daily from different units of the CTU. It really gave me a lot of knowledge.”- FGD3.*

### Learning various Oxygen administration modalities

*“I did not personally know all the modalities of oxygen administration but through this experience, I learned how to administer oxygen using the different modalities.” -FGD 2*.

*“ Before it was only the doctors requesting for tests, but we now learned that even nurses can use these tests to identify and manage conditions like hyperkalemia. Up to date, we use the same knowledge on these other wards to manage patients.”- FGD2*.

### Learning critical care management of patients

*“I really learned a lot in critical care and became so passionate about it. I am in critical care, and I am passionate about critical care. These skills motivated me to continue pursuing a career in critical care.”- FGD 1*.

*“ It was a good experience that equipped us with a lot of knowledge in terms of pulmonology and interpretation of the X-ray. You were able to diagnose and see where the pathology is.”-FGD2*.

To some, the skills learned enabled them to obtain new job positions in other organizations during the different phases of COVID-19 or even after the pandemic was over.

*“The experience I got in COVID_19 also helped me to get a job in the medical research council (another facility) where I was employed to take samples of patients with COVID-19.”- FGD 2*.

## Discussion

This study aimed to establish the experiences of nurses who managed COVID-19 patients at the largest COVID-19 treatment hospital in Uganda. The fact that COVID-19 was characterized by long hospitalization with an average of 20.6 days [19], meant that a lot of nursing care went into ensuring patients’ recovery from the disease. Uganda is one of the countries that did fairly well in managing the COVID-19 pandemic despite being a resource-limited setting with a 98% recovery rate [4].

The nurses provided a range of services to the admitted patients which included drugs, oxygen and fluid administration, nutritional care, intense monitoring of patient vital signs, positioning and turning of patients, maintaining overall hygiene of the patient, and psychological support. These are similar to the various COVID-19 nursing interventions in other parts of the world [26]. However, the frequency of vital sign monitoring among COVID-19 patients in Uganda was much higher than that reported by Asiimwe et al. among clients battling with severe critical illnesses in general wards within the same country [7]. An England-based study identified that 31% of the preventable deaths in hospitals were attributed to poor vital monitoring [8] and therefore the trend of monitoring seen in this study was ideal in the timely identification of hemodynamically unstable patients and hence abated mortality.

Although COVID-19 was a frightening disease that led to extreme levels of stress among the nurses, nurses in this study highlighted a sense of obligation to work as they cared for the COVID-19 patients. A similar experience was reported by frontline nurses in Iran, Kenya, and Ghana [3, 10, 15]. This could be attributed to the nurses considering their profession as a calling to serve, exercise selflessness, and give back to their country and thus a source of professional satisfaction.

Uganda’s decision to provide financial incentives for frontline nurses was an approach that had been implemented by other countries during the pandemic [20, 21]. Our findings showed that financial incentive provided to the frontline nurses that were treating the COVID-19 patients was an important motivator for them to continue rendering services to the clients. This means that financial incentives lead to greater job satisfaction and job sacrifices for the nurses hence impacting the quality of care received and treatment patterns in a positive way as seen in other studies [22, 23]. This study finding is consistent with that reported by the frontline line health workers in Ghana and Japan [22, 23]. Similarly, a study conducted in India also reported that besides safety measures, health professionals require financial incentives to battle at the front lines to defeat pandemics [24].

Greater interprofessional collaboration especially with seniors across the various healthcare professionals was reported among the nurses in this study as one of the most significant changes that came along with the management of the COVID-19 patients. This translated to improved learning, creativity, psychological safety, and communication among the interprofessional healthcare teams which formed a basis for supportive networks and timely management of clients through faster decision-making [25, 26]. The teamwork and solidarity experienced by the nurses in Uganda who managed COVID-19 patients at Mulago National Referral Hospital were consistent with the findings experienced by other nurses across the globe [27–29]. Even at the end of the pandemic, nurses desired that the interprofessional collaboration experienced during the COVID-19 pandemic response be sustained.

Similar to other countries, the healthcare systems in Uganda became overwhelmed with a serious shortage of nurses, and the few available ones performed more tasks. Despite the government beefing up the existing human resources through the recruitment of contract nurses, the number remained low with at least each nurse serving five patients in the intensive care unit. The 1:5 nurse-patient ratio reported by nurses in ICU was way above the WHO-recommended one of 1:2 [30] but consistent with the findings of > 1:2 in the survey done by Atumanya et al. in 2020 in Uganda [29]. Participants expressed the fact that the situation became pathetic particularly at the peak of the pandemic when patients’ numbers doubled increasing the strain on the few that were available due to the poor nurse-patient ratio. In line with recent studies related to COVID-19 care, this study also found that work overload led to physical and psychological distress among nurses [20]. Burnout due to work overload was also reported by the majority of the participants and this was consistent with the findings by Nishimura et al. and Kabunga et al. where 30–50% of healthcare workers experienced burnout during the COVID-19 pandemic [20].

Unlike other studies where nurses reported inadequate responses from the hospital and local authorities concerning the establishment of protocols, guidelines, isolation wards, and management of PPE shortages [14, 15, 30], nurses in this study have commended the role played by the hospital management and government authorities in guiding the public and providing PPE. Furthermore, while nurses in countries like Nigeria reported feeling underappreciated and under-valued by their government [15], this study revealed that the Uganda government, led by the president himself appreciated the tremendous work done by the nurses as evidenced in the public recognition as “essential workers” that are at the frontline of fighting COVID-19. This showed how valuable nursing services are not only during the pandemic but also to humanity [31]. This is similar to findings from the Arabian Gulf where some people perceived these nurses as heroes fighting the disease and protecting society [13]. This kind of appreciation promoted their satisfaction and boosted their self-confidence.

One positive aspect that was widely reported among nurses in this study was professional development through acquiring new practical knowledge and skills in pandemic and highly infectious diseases nursing care. These among others and just like in other studies included; knowledge and skills in triage criteria, transport of patients to intensive care, nasopharyngeal swab and nasopharyngeal aspirate (NPA) procedures, safe handling of test specimens, and transfer and admission of suspected or confirmed cases under quarantine [32]. This, therefore, means that their experience in the management of COVID-19 patients improved their readiness and built confidence in infection prevention and control and their ability to take care of patients with COVID-19 [13, 33].

Generally, a strong professional commitment to serving COVID-19 patients is exhibited in this study by the nurses. However, the findings also highlight the need for physical, psychological, and spiritual support in boosting the morale of nurses and other health workers. Political and government support was key in enabling the nurses to persevere as they cared for the COVID-19 patients.

## Conclusion

The nurse’s experiences can be described as those characterized by resilience and professional commitment to serve despite negative scenarios like work burnout due to high workload, social isolation, and psychological stress. Despite the hardships the nurses faced in the management of COVID-19 patients, interprofessional teamwork, financial incentives, strong political and government support, provision of PPE, and resources to use by the hospital were some of the key motivators for nurses to provide care to COVID-19 patients. Their experiences enabled them to gain new knowledge and skills in pandemic and highly infectious disease management given that most of them hardly had any prior experience in epidemic and pandemic management.

### Recommendations

For future research, a Delphi study to enable the development of COVID-19 nursing clinical guidelines would be useful to advance adequate management of the disease in the day-to-day cases in Uganda, Africa, and globally. For practice and policy to enhance response to future epidemics and pandemics, it is key to have effective systems in place to support the well-being of the nurses and other health workers.

### Limitations

This study was qualitative and thus prone to participant acquiescence and recall bias. However, the research team ensured open-ended questions and provided enough time to provide in-depth responses with an emphasis on the correct understanding of the questions by the participants. The study was only conducted in Mulago National Referral Hospital which may be seen as a weakness however, this was the hospital that managed the most cases of COVID-19 patients and had the most nurses and other health workers participating in care. The strength of the results is that they are well triangulated capturing experiences from nurses of various professional education and seniority in the profession. There was no major difference in the findings despite the various professional education and seniority in the profession.

### Electronic supplementary material

Below is the link to the electronic supplementary material.

Supplementary Material 1

## Data Availability

The tools and data set for this study are available upon reasonable request from the corresponding author.

## References

[1] Myers LC, Liu VX (2022). The COVID-19 pandemic strikes again and again and again. JAMA Netw Open.

[2] Moyo I. Nurses’ experiences of providing care to suspected COVID-19 patients in a resource limited setting. Cogent Public Health. 2022;9.

[3] Sun S, Xie Z, Yu K, Jiang B, Zheng S, Pan X (2021). COVID-19 and healthcare system in China: challenges and progression for a sustainable future. Globalization and Health.

[4] World Health Organization. WHO, Coronavirus Disease. (COVID-19) Dashboard With Vaccination Data | WHO Coronavirus (COVID-19) Dashboard With Vaccination Data. World Health Organization. 2023;:1–5. https://covid19.who.int/%0Ahttps://covid19.who.int/%0Ahttps://covid19.who.int/region/searo/country/bd. Accessed 13 Apr 2023.

[5] Wise J (2023). Covid-19: WHO declares end of global health emergency. BMJ.

[6] Siemieniuk RAC, Bartoszko JJ, Ge L, Zeraatkar D, Izcovich A, Pardo-Hernandez H (2020). Drug treatments for covid-19: living systematic review and network meta-analysis. The BMJ.

[7] Marcolino MS, Meira KC, Guimarães NS, Motta PP, Chagas VS, Kelles SMB (2022). Systematic review and meta-analysis of ivermectin for treatment of COVID-19: evidence beyond the hype. BMC Infect Dis.

[8] De Crescenzo F, Amato L, Cruciani F, Moynihan LP, D’Alò GL, Vecchi S et al. Comparative effectiveness of pharmacological interventions for Covid-19: a systematic review and network Meta-analysis. Front Pharmacol. 2021;12 May. 10.3389/fphar.2021.649472 PMC8126885 34012398

[9] Kirenga B, Byakika-Kibwika P, Muttamba W, Kayongo A, Loryndah NO, Mugenyi L (2021). Efficacy of convalescent plasma for treatment of COVID-19 in Uganda. BMJ Open Respiratory Research.

[10] Belayneh A (2020). Off-label use of Chloroquine and Hydroxychloroquine for COVID-19 treatment in Africa Against WHO recommendation. Res Rep Trop Med.

[11] Fawaz M, Anshasi H, Samaha A (2020). Nurses at the Front line of COVID-19: roles, responsibilities, risks, and rights. Am J Trop Med Hyg.

[12] Roberts NJ, Kelly CA, Lippiett KA, Ray E, Welch L (2021). Experiences of nurses caring for respiratory patients during the first wave of the COVID-19 pandemic: an online survey study. BMJ Open Respiratory Research.

[13] Nasaif H, Aldiabat K, Alshammari M, Albloushi M, Alblooshi SM, Yaqoob S. The lived experiences of nurses caring for patients with COVID-19 in Arabian Gulf Countries: a Multisite Descriptive Phenomenological Study. Global Qualitative Nursing Research. 2023. 10. 10.1177/23333936231155052 PMC9969219 36855739

[14] Chau JPC, Lo SHS, Saran R, Leung CHY, Lam SKY, Thompson DR (2021). Nurses’ experiences of caring for people with COVID-19 in Hong Kong: a qualitative enquiry. BMJ Open.

[15] Popoola T, Popoola V, Nelson K. Nurses’ lived experiences of caring for patients with COVID-19 in Nigeria. SAGE Open Nursing. 2022;8. 10.1177/23779608221117384 PMC9364185 35966231

[16] Rathnayake S, Dasanayake D, Maithreepala SD, Ekanayake R, Basnayake PL (2021). Nurses’ perspectives of taking care of patients with coronavirus disease 2019: a phenomenological study. PLoS ONE.

[17] Sepeng NV, Makhado TG, Makhado L. Conceptual Framework for rape survivors diagnosed with PTSD in the North West Province of South Africa. Healthc (Switzerland). 2023;11. 10.3390/healthcare11010127 PMC9819654 36611586

[18] Mwine P, Atuhaire I, Ahirirwe SR, Nansikombi HT, Senyange S, Elayeete S (2023). Readiness of health facilities to manage individuals infected with COVID-19, Uganda, June 2021. BMC Health Serv Res.

[19] Kirenga B, Muttamba W, Kayongo A, Nsereko C, Siddharthan T, Lusiba J (2020). Characteristics and outcomes of admitted patients infected with SARS-CoV-2 in Uganda. BMJ Open Respir Res.

[20] Kabunga A, Okalo P (2021). Prevalence and predictors of burnout among nurses during COVID-19: a cross-sectional study in hospitals in central Uganda. BMJ Open.

[21] Exploratory Research Design in Management Science. : A Review of Literature on Conduct and Application. International Journal of Research and Innovation in Social Science. https://www.rsisinternational.org/journals/ijriss/articles/exploratory-research-design-in-management-science-a-review-of-literature-on-conduct-and-application/. Accessed 4 Sep 2023.

[22] Bongomin F, Fleischer B, Olum R, Natukunda B, Kiguli S, Byakika-Kibwika P (2021). High Mortality during the Second Wave of the Coronavirus Disease 2019 (COVID-19) pandemic in Uganda: experience from a National Referral COVID-19 Treatment Unit. Open Forum Infectious Diseases.

[23] Saunders B, Sim J, Kingstone T, Baker S, Waterfield J, Bartlam B (2018). Saturation in qualitative research: exploring its conceptualization and operationalization. Qual Quant.

[24] Braun V, Clarke V (2006). Using thematic analysis in psychology. Qualitative Res Psychol.

[25] UMEA University. Open Code 4.03. 2015. https://www.umu.se/en/department-of-epidemiology-and-global-health/research/open-code2/. Accessed 5 Apr 2022.

[26] Asghari E, Archibald M, Roshangar F (2022). Nursing interventions for patients with COVID-19: a medical record review and nursing interventions classification study. Int J Nurs Knowl.

[27] Gavric G, Vesic T, Novakovic N. The importance of financial incentives for healthcare workers during the Covid 19 pandemic. Bizinfo Blace. 2023;14.

[28] Morishita K, Katase K, Ishikane M, Otomo Y. Motivating factors for frontline healthcare workers during the COVID-19 pandemic: a survey in Japan. Curr Psychol. 2022;:1–9. 10.1007/s12144-022-04177-6 PMC9803592 36618542

[29] Atumanya P, Sendagire C, Wabule A, Mukisa J, Ssemogerere L, Kwizera A (2020). Assessment of the current capacity of intensive care units in Uganda; a descriptive study. J Crit Care.

[30] Irandoost SF, Yoosefi Lebni J, Safari H, Khorami F, Ahmadi S, Soofizad G (2022). Explaining the challenges and adaptation strategies of nurses in caring for patients with COVID-19: a qualitative study in Iran. BMC Nurs.

[31] Firouzkouhi M, Abdollahimohammad A, Rezaie-kheikhaie K, Mortazavi H, Farzi J, Masinaienezhad N. Nurses’ caring experiences in COVID-19 pandemic: A systematic review of qualitative research. Health Sciences Review. 2022;3 May. 10.1016/j.hsr.2022.100030 PMC9123825 35615410

[32] Chau JPC, Lo SHS, Saran R, Leung CHY, Lam SKY, Thompson DR (2021). Nurses’ experiences of caring for people with COVID-19 in Hong Kong: a qualitative enquiry. BMJ Open.

[33] Sun N, Wei L, Shi S, Jiao D, Song R, Ma L (2020). A qualitative study on the psychological experience of caregivers of COVID-19 patients. Am J Infect Control.

