# Supplementary material for: “We felt so proud by the president calling us my heroes.” An exploration of the nurse’s experiences in the management of COVID-19 patients in Uganda
Source: BMC Nurs. 2023 Oct 3;22:352. doi: 10.1186/s12912-023-01503-6 (PMC10548748; doi:10.1186/s12912-023-01503-6)
Supplement: Supplementary file 1 — Supplementary Material 1 [file 12912_2023_1503_MOESM1_ESM.docx]

**Appendix 1**

**FGD interview Guide for establishing the experiences of Nurses Caring for COVID 19 Patients in Uganda.**

**Social Demographic Characteristics**

Participant ID # __________ Hospital ID #_________________

Age: ____________ Gender: ____________

Marital Status: _________________ Number of children or dependents: _____________

RN _______/RM _______ Total years of working experience: __________

Educational level: Diploma______ Certificate_____ BSc. _____ MSC/MPH _____ PhD _____

Religious affiliation: _________________

Did you have access to personal protective equipment: Yes________ No _

Have you worked in highly infectious diseases wards before? If yes which clinical conditions?

**COVID 19 Nursing Care provided**

What kind of nursing care did you provide to the patients?

- Medical care,
- Nutritional care,
- psychosocial care
- Any other type of care

How long on average were COVID 19 patients admitted by the time of discharge?

**Experiences of the Nurses Providing COVID 19 Care**

1. What were your experiences during the COVID19 outbreak in Uganda?

2. What made you decide to work on the COVID 19 ward?

3. What resources did you draw on to help you handle the situation?

4. Are there other ways you think you might have handled the situation under

similar circumstances?

5. How did you feel about working in a COVID 19 ward and nursing COVID19 patients?

6. In what ways did your personal resources contribute to influencing your

willingness to work during the COVID 19 crisis?

7. In what ways did your institution or hospital influence your willingness to

work on the COVID 19 ward during the crisis?

8. In what ways did the political climate (e.g. news reports, television, national

leaders) influence your willingness to work during the crisis?

9. In what ways did the government (local and national) influence your

willingness to work during the crisis.

10. Which one of these resources was most influential and why?

11. What were your thoughts as you received and cared for patients in the COVID 19 ward?

12, How did your experiences or thoughts and feelings during the COVID 19 pandemic affect how you received and care for patients?

13. were you able to interact with your family as you carried out this work? In other words, did you go back home daily after your shift?

where are your family members freely interacting with you? if not why and how did it make you feel?

14. Did Family members discourage you to work?

15. When family members discouraged you to work, how did you respond?

16. How did you feel and react to them and your work?

17. At work you had to be dressed up in PPE, tell us more about your experience having to wear PPE full time

18, When nurses were no longer touching each other, how did you feel? How

did it affect you professionally? What other measures do you think could

you have taken?

19. What did you gain from your experience personally? Professionally?

20. What lessons did you learn from your experience?

Adapted from (Kollie, 2016)

Kollie, E. (2016). Experiences of Nurses and Midwives during the Ebola Outbreak in Liberia, West Africa. *Loma Linda University Electronic Theses, Dissertations & Projects*. https://scholarsrepository.llu.edu/etd/384
